# In Situ Monitored Vortex Fluidic-Mediated Protein Refolding/Unfolding Using an Aggregation-Induced Emission Bioprobe

**DOI:** 10.3390/molecules26144273

**Published:** 2021-07-14

**Authors:** Qi Hu, Haozhen Hu, Xinyi Zhang, Kyle Fan, Yuning Hong, Colin L. Raston, Youhong Tang

**Affiliations:** 1 Medical Device Research Institute, College of Science and Engineering, Flinders University, Adelaide, SA 5042, Australia; qi.hu@flinders.edu.au (Q.H.); haozhen_hu@126.com (H.H.); xinyi.zhang@flinders.edu.au (X.Z.); 2 Flinders Institute for Nanoscale Science and Technology, College of Science and Engineering, Flinders University, Adelaide, SA 5042, Australia; kfan6911@alumni.sydney.edu.au (K.F.); colin.raston@flinders.edu.au (C.L.R.); 3 Department of Chemistry and Physics, La Trobe Institute for Molecular Science, La Trobe University, Melbourne, VIC 3086, Australia; Y.Hong@latrobe.edu.au

**Keywords:** vortex fluidic device, protein folding/unfolding, aggregation induced emission

## Abstract

Protein folding is important for protein homeostasis/proteostasis in the human body. We have established the ability to manipulate protein unfolding/refolding for β-lactoglobulin using the induced mechanical energy in the thin film microfluidic vortex fluidic device (VFD) with monitoring as such using an aggregation-induced emission luminogen (AIEgen), TPE-MI. When denaturant (guanidine hydrochloride) is present with β-lactoglobulin, the VFD accelerates the denaturation reaction in a controlled way. Conversely, rapid renaturation of the unfolded protein occurs in the VFD in the absence of the denaturant. The novel TPE-MI reacts with exposed cysteine thiol when the protein unfolds, as established with an increase in fluorescence intensity. TPE-MI provides an easy and accurate way to monitor the protein folding, with comparable results established using conventional circular dichroism. The controlled VFD-mediated protein folding coupled with in situ bioprobe AIEgen monitoring is a viable methodology for studying the denaturing of proteins.

## 1. Introduction

In the human body, protein folding is the process whereby a protein obtains its functional structure and conformation. Through this physical process, proteins fold from irregular folding to a specific functional three-dimensional structure. Eventually, the body reaches a state called proteostasis which represents a balance between the folding of newly synthesized protein and removal of misfolded proteins [1]. While in some cases such as gene mutation, the proteins can misfold and aggregate, and finally break the state of proteostasis. The aggregation of misfolded proteins can form ordered structures like amyloid fibrils. The diseases associated with amyloids are known as amyloidosis, with common amyloidosis including Alzheimer’s disease, Parkinson’s disease, and Huntington’s disease [2,3,4]. More importantly, the clarification of the protein folding mechanism that represents its theoretical significance is an essential pathway to naturally reveal the genetic codes in different species. The research of protein folding, in a narrower sense, is to study the formation of a specific three-dimensional structure of a protein, its stability, and its relationship with its biological activity. Consequently, protein folding is a crucial indicator, reflecting physiological conditions on the level of protein molecules, to investigate and predict the states of amyloidosis indirectly.

The vortex fluidic device (VFD) can generate a dynamic thin fluid film to rapidly and efficiently fold proteins by providing high shear stress, high shearing rates, rapid heat and mass transfer, and intense micro-mixing [5,6,7]. This is understood as arising from different topological fluid flows down to submicron dimensions [6]. At a tilt angle of 45° for the tube in the VFD, the Coriolis force from the hemispherical base of the tube generates a spinning topological top flow with Faraday waves generating eddies across the thin film which are twisted into double helices by the Coriolis force from the curved walls of the tube. This double helical topological flow can combine with the spinning top of the same diameter, creating spicular or spheroidal topological fluid flow. The high mass transfer and heat transfer from these topological fluid flows, for which their presence depends on the nature of the liquid and the rotational speed of the tube, can be harnessed to control chemical transformations [8,9], accelerate protein folding [10,11,12] and bio-catalysis, prepare materials synthesis and probe self-organised systems [13,14]. The Ig Nobel chemistry prize, ‘A chemical recipe to partially unboil an egg’ in 2015, achieves refolding of proteins in the VFD, which has potential for protein renaturation [15] and this is explored further herein. The aforementioned applications of the VFD are optimal at a tilt angle of 45°, and this was the choice of tilt angle in the present study.

To date, various methods, including circular dichroism (CD) and fluorescence-based methods, have been used for protein folding detection and each of these methods has its advantages and disadvantages. To be more specific, CD is a useful technique when measuring protein secondary structure and protein tertiary structure for structure loss upon denaturation [16,17,18,19]. The principle is that it emits circularly polarized light, with optically active chiral molecules having preferential absorbance for one direction of circularly polarized light. Due to different absorption of a protein to left-hand circular polarized light and right-hand circular polarized light, the CD spectra of the protein can be shown as ellipticity over a range of wavelength where the information on protein secondary structure is uniquely described [20,21,22]. As in the fluorescence methods, aggregation-induced emission (AIE) sensing strategies have excellent performance on studying protein unfolding/refolding states [23,24,25,26,27]. Tetraphenylethene maleimide (TPE-MI) consists of the AIE fluorogen tetraphenylethane (TPE) and the thiol-reactive group maleimide (MI), thereby possessing both AIE phenomenon and selective thiol reactivity [28]. According to our previous research [28], TPE-MI is non-emissive, even on conjugating with free thiols in aqueous media, but can produce fluorescence when it is bound with cysteine residues exposed from folded proteins. This is because the intramolecular motion of TPE core is unable to be constrained sufficiently by small and soluble thiols. The excitation and emission wavelengths are 350 nm and 470 nm respectively, which is a large Stokes shift. The hypothesis herein is that cysteine is the least surface exposed amino acid in a correctly folded protein but when the protein starts to unfold, the cysteine becomes more exposed whereupon the MI group will react with the cysteine to form a covalent bond. As such the resulting fluorescence intensity will directly determine the amount of unfolded protein [29,30,31]. In contrast to the CD method, AIE fluorescence is more affordable, and the TPE-MI allows monitoring of the protein unfolding directly, thus with the ability target these thiols as indicators for the level of unfolded proteins [32].

In this work, the VFD thin film microfluidic platform is used to manipulate protein unfolding/refolding as a contribution to control and monitor protein denaturation/renaturation. On the basis of the low molecular weight and comparatively simple structure, β-lactoglobulin was the protein of choice in the present study, with the processing also in the presence of a high concentration of the chaotropic agent guanidine hydrochloride (GuHCl), which is a well-known denaturant of proteins. These were applied in combination to accomplish the protein unfolding state with the protein structure detection and folding monitored using the novel AIEgen, TPE-MI, with the folding rad out form this method verified using conventional CD. Combining VFD and AIE technologies provides a fully capable and robust method for manipulating and monitoring the progress of protein denaturation and renaturation.

## 2. Experimental Section

### 2.1. Materials

The materials used in this study include the protein β-lactoglobulin, denaturant guanidine hydrochloride (GuHCl), dimethyl-sulfoxide (DMSO) and phosphate-buffered saline (PBS), all purchased from Sigma-Aldrich (Sydney, Australia). Water was purified by a filtration system (Millipore, Adelaide, Australia). The AIE fluorescence dye TPE-MI was synthesised according to our previous publication [29]. All the experiments were performed at room temperature unless otherwise specified.

The solid AIEgen, TPE-MI was dissolved in DMSO as stock solution at a concentration of 50 μM. The stock solution was kept at 4 °C in the dark for long term storage. Furthermore, the recommended working solution of TPE-MI was prepared daily by suitable dilution of the stock solution with PBS buffer (pH = 7.4). For each set of experiments, 10 mL of TPE-MI working solution was prepared and placed under room temperature for 1 h before use. Protein β-lactoglobulin was powder-like and was stored at 4 °C prior to use, whereupon it was dissolved in the PBS buffer (pH = 7.4) at 50 μM solution which was allowed to reach room temperature [33].

### 2.2. Characterisations

Fluorescence spectrometer (Cary Eclipse, Melbourne, Australia) was turned on for 15 min before testing, using then an excitation wavelength and emission wavelength of TPE-MI, 350 nm and 470 nm, respectively. The emission mode was set in the wavelength range of 400 nm to 600 nm. The sample volume within the cuvette was 3 mL. CD spectrophotometer (Jasco, Tokyo, Japan) was flushed with nitrogen at least 5 min before using, with the nitrogen flow rate strictly followed for different ranges of wavelength. The sample volume within the cuvette was 250 μL. The vortex fluidic device (VFD) from the Flinders University College of Science and Engineering was employed. There was no preheating requirement for VFD, but in order to maximize the effect of the shear stress with the thin film extending close to the top of the tube, 2 mL liquid in a 10 mm OD VFD tube (8.5 mm ID, 18.5 cm in length), the parameters of VFD for this study were 5000 rpm rotational speed, 45° tilt angle, clockwise (CW) rotation (looking down the tube) and 10 min operating time. The choice of 5000 rpm rotational speed was based on the optimised success as such for earlier folding experiments in the 10 mm OD VFD tube [7].

### 2.3. Protocol Development

**Figure 1:**
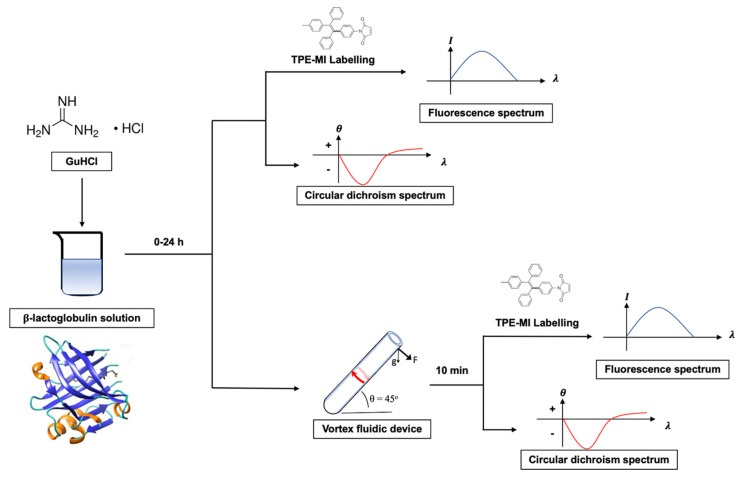
Methodology of manipulating and monitoring protein unfolding/refolding.

The strategy for the experiments is shown in Figure 1. The guanidine hydrochloride (GuHCl) was added to the prepared β-lactoglobulin solution in a ratio of 1:4 (*v*/*v*, mL). The amount of denaturant was less than the amount of protein added to make sure that all of denaturant was fully capable of being consumed at the end of the reaction. Moreover, the incubation time of up to 24 h was evaluated where a dynamic denaturing progress was able to be conducted during this long period. The fluorescence intensity of TPE-MI was used to monitor real-time the extent of denaturation, with CD used for verification post VFD processing. Based on the previous step, VFD was introduced to manipulate protein after and during the progress of denaturation.

## 3. Results and Discussion

### 3.1. Kinetic Optimization

The strong nucleophilic property of the thiol group plays a significant role in TPE-MI targeting. However, thiol-based reactions that occur under normal conditions depend on labelling time. β-lactoglobulin has only one free cysteine and four cysteines that form disulfide bonds. Initially, TPE-MI is non-emissive in aqueous media, attributed to the exciton annihilation process associated with the n-*π* electronic conjugation of the carbonyl and olefinic groups. When the thiol is introduced from denatured β-lactoglobulin, a thiol-ene hydrothiolation reaction will inevitably happen. The attraction of the MI moiety will conjugate the thiol group to its C=C double bond where n-*π* conjugation is broken and the AIE effect is recuperated [28]. When the reaction approaches the end point, the fluorescence intensity will no longer increase, and the combination of thiol and MI groups has become saturated, therefore, all the unfolded proteins have produced fluorescence effects. Theoretically, the reaction ratio between TPE-MI and β-lactoglobulin is 1/1. This methodology can be used here to evaluate the kinetic of AIE labelling of the unfolded protein and the denaturant concentrations optimized for protein unfolding.

**Figure 2:**
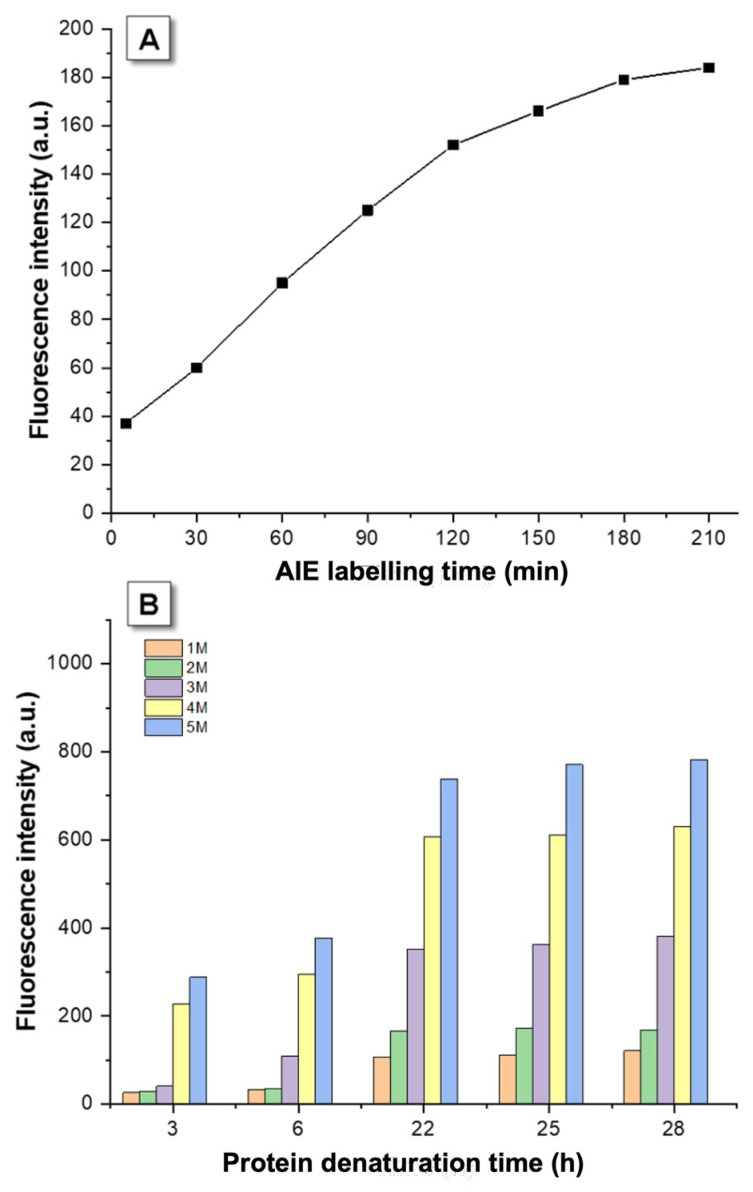
Kinetic experiments during AIE labelling and denaturant concentration optimization. (**A**) The time of AIEgen TPE-MI labelling with GuHCl denaturant (2 M), and (**B**) Fluorescence intensity changes with respect to reaction time with 1–5 M denaturant concentrations of GuHCl. The fluorescent intensity measurements were carried out in PBS buffer with pH = 7.4; [TPE-MI] and [β-lactoglobulin] = 50 μM, λ_ex_ = 350 nm.

Figure 2A shows the fluorescence intensity variation during 210 min of AIE labelling after denaturant (2 M GuHCl) was reacted with the protein for a long denaturation time, over 24 h, ensure that the denaturant had been completely consumed and no further unfolded protein was produced during the labelling process. The intensity increased gradually in the first two and a half hours then tended to be stable after 3 h, even though it still increasing slightly. Thus, TPE-MI would complete the time-dependent labelling with the unfolded β-lactoglobulin at 3 h and the optimal labelling time of TPE-MI and thiol reaction set as 3 h in this study.

The equilibrium of denaturation is a dynamic process where natural and unfolded proteins convert to each other as a free energy process [34] and the protein folding kinetics is highly correlated with denaturant time and concentration. In the present study the kinetic experiment examines unfolding by monitoring changes in a fluorescent signal over time following the initiation of the reaction. Denaturation times of 3, 6, 22, 25 and 28 h were used to determine the denaturation performance for different concentrations of 1 M, 2 M, 3 M, 4 M and 5 M GuHCl, respectively (Figure 2B). After 24-h of denaturation, the fluorescence intensities of different concentrations tended to be stable, and hence the denaturation reaction was considered finished after 24 h. In addition, the results show that higher concentrations of GuHCl results in higher fluorescence intensity over 24 h denaturation, suggesting that for each point, there is still undenatured protein. The reactions stop because of depletion of the denaturant.

In the process of denaturation, the rising fluorescence intensity indicates that proteostasis is located in the intermediate state of denaturation, within 3 to 22 h. Based on this, denaturation times of 3 h and 24 h were selected for further studies as the intermediate state and the denatured state respectively. Notably, for the purpose of avoiding overrange of the fluorescence intensity in further studies, 4 M GuHCl was chosen as the appropriate denaturation concentration. To be more specific, two protein situations were chosen for further studies, namely (1) refolding protein using VFD processing where the denaturation process was finished after 24 h, with no additional denatured protein subsequently produced, and (2) the denaturation process was still occurring (3 h denaturation) and could then be accelerated using VFD processing.

### 3.2. AIEgen Monitoring Protein Denaturation

**Figure 3:**
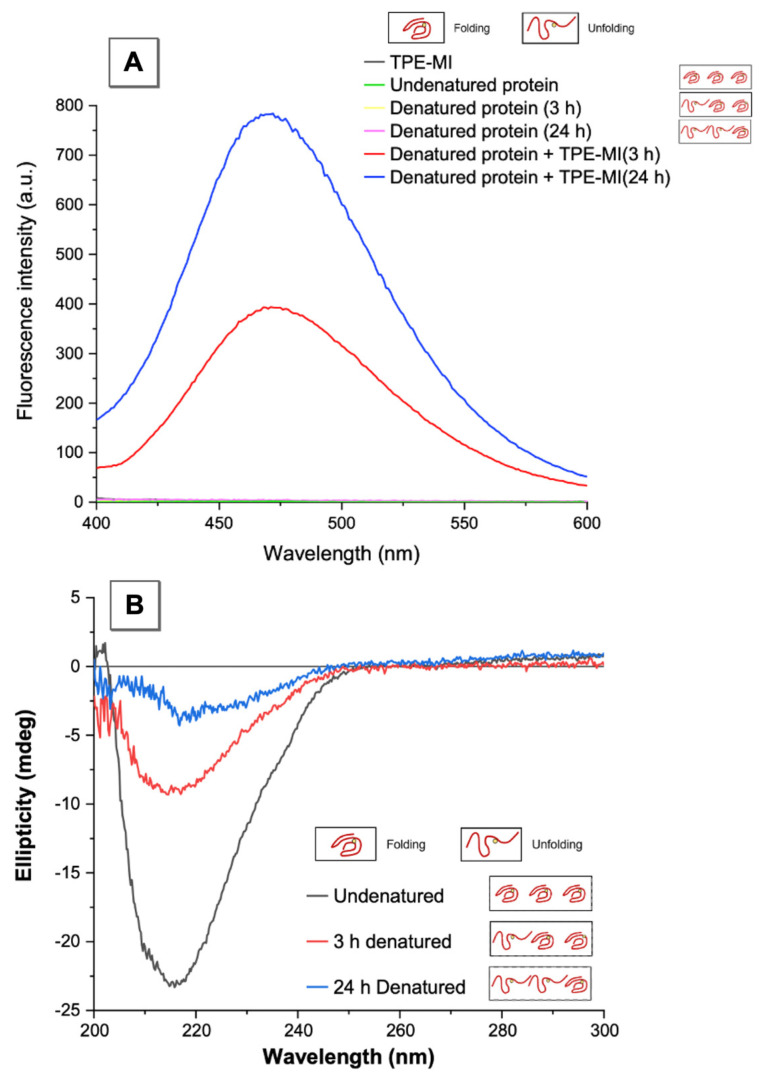
Fluorescence spectra and circular dichroism spectra for identifying the protein denaturation state. (**A**) Fluorescence spectra of TPE-MI, undenatured protein, 3 h and 24 h denatured protein, 3 h denatured protein with TPE-MI and 24 h denatured protein with TPE-MI. (**B**) The CD spectra of undenatured protein, 3 h denatured protein and 24 h denatured protein. The spectra detections were carried out in PBS buffer with pH = 7.4. [TPE-MI] and [β-lactoglobulin] = 50 μM, [GuHCl] = 4 M. λ_ex_ = 350 nm (fluorescence), λ_peak_ = 217 nm (CD).

AIEgen, TPE-MI was used to monitor protein denaturation, with CD used to verify the results. Figure 3A shows fluorescence intensity change when TPE-MI was used to monitor the denatured protein, with no signal on the emission wavelength at the beginning, but with a distinct fluorescence peak at 470 nm appearing after 3 h and 24 h denaturation respectively, arising from the reaction between denatured β-lactoglobulin and TPE-MI. According to the AIE, the denaturation times (3 h and 24 h) established an abundance of unfolded proteins. Figure 3A establishes that the amount of unfolded β-lactoglobulin is linked to the fluorescence intensity. Here the higher fluorescence intensity, the more unfolded protein, and thereby the pathway through fluorescence intensity demonstrates that the protein transforms from the native state to the denatured state.

CD is employed to identify the protein denaturation, as well as to further validate the fluorescence method developed herein [34]. The protein used as such, β-lactoglobulin, is a typical β-sheet predominant protein, where a minimum 217 nm peak represents characteristic of β-sheet content [35,36,37]. In Figure 3B, the shape of peak provided insight into the nature of the secondary structure, which was primarily composed of β-sheets. With the ellipticity increasing related to denaturing progress, the protein secondary structure tended to transform from β-sheet to a random coil conformation, indicating that the destruction of protein homeostasis could promote the unfolding of the protein, and further lead to acceleration of protein aggregation. After 24 h denaturation, the ellipticity of β-lactoglobulin at 217 nm was lowest at negative 3 millidegrees, which reduced by 86.9% compared to initial one. This established that the larger the ellipticity, i.e., less β-sheet content, the more unfolded β-lactoglobulin was present. Consequently, the CD spectrum illustrates the change of β-lactoglobulin structure, from folding to unfolding, and also it effectively validates that AIEgen, TPE-MI, accurately monitors the protein denaturation.

### 3.3. VFD Manipulating Protein Refolding/Unfolding

**Figure 4:**
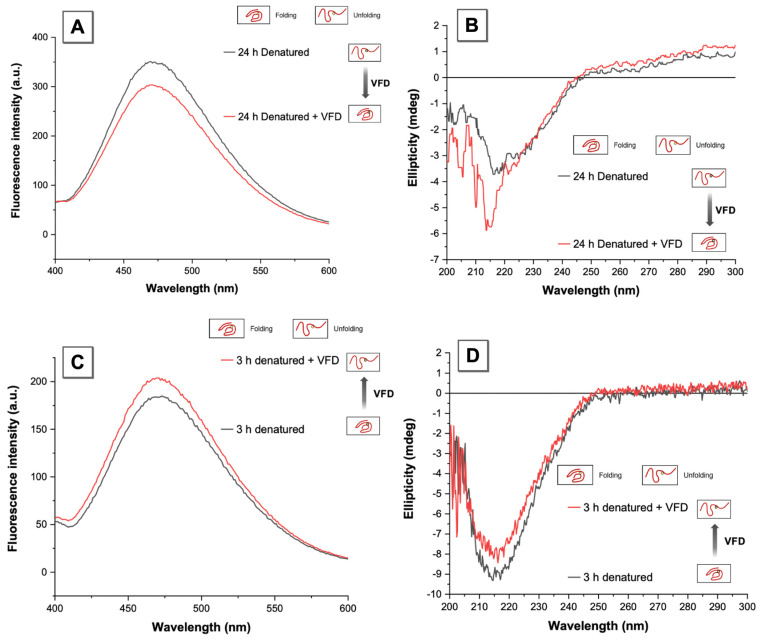
Fluorescence and CD spectra for protein refolding/unfolding using VFD processing. (**A**) Fluorescence intensity change and (**B**) Ellipticity change of the protein after 24 h of denaturation with or without 10 min of VFD processing. (**C**) Fluorescence intensity change, and (**D**) Ellipticity change of the protein after 3 h of denaturation with and without 10 min of VFD processing. The spectra were recorded in PBS buffer at a pH = 7.4. [TPE-MI] and [β-lactoglobulin] = 50 μM, [GuHCl] = 4 M. λ_ex_ = 350 nm (fluorescence), λ_peak_ = 217 nm (CD).

Following the successful monitoring of the fluorescent protein in different denaturation states, which was validated using CD, the effect of VFD towards the degree of protein denaturation was investigated, focusing on two marked states, the denatured state (24 h) and the intermediate state (3 h). Here, TPE-MI was added and incubated for 3 h to ensure the AIE dye was fully labelled on the denatured proteins (24 h in GuHCl) prior to fluorescence measurements (Figure 4A). The emission wavelength of TPE-MI labelled protein was at 470 nm, as previously reported, with the fluorescence of the 24 h denatured protein with TPE-MI around 350 a.u., i.e., the decrement of fluorescence intensity due to VFD processing was relatively stable at around 50%, reducing 14% of the total intensity, which could be regarded as the refolding efficiency. As the previous CD spectrum indicated (Figure 3B), for the protein β-lactoglobulin, for denaturation induced by GuHCl, the bigger the ellipticity at 217 nm, the higher the extent of denaturation, i.e., with less β-sheet content. In Figure 4B, the ellipticity decreased by more than 2 millidegrees after VFD processing, compared to >−4 millidegrees of the total ellipticity of the denatured protein, and thus incremental change in refolding was significant. This ability to refold proteins using the VFD was in accord with previous studies [7]. Figure 4C,D show the opposite effect; instead of protein refolding after denaturation, the VFD promoted the denaturation process in the presence of GuHCl, resulting in bigger ellipticity at 217 nm. Similarly, the fluorescence result in Figure 4C indicates that VFD processing during denaturation promoted β-lactoglobulin unfolding. This arises because the protein denaturation reaction is a dynamic and reversible process. For the intermediate processing time (3 h), the presence of a high concentration of chaotropic agent GuHCl and the high shear stress produced in the thin film of liquid in the VFD promotes the formation of more unfolded proteins. On the contrary, when all proteins are unfolded in the presence of GuHCl after 24 h, the high shear stress in the VFD forces the proteins to refold, generating correctly folded protein. Therefore, the refolding and protein unfolding process is always synchronized. Under different denaturation states and conditions, one of the two will be dominant with the VFD processing a simple and useful technology to manipulate folding states.

### 3.4. Interpretation of Equilibrium of Unfolding and Refolding Proteins by VFD and AIEgen

**Figure 5:**
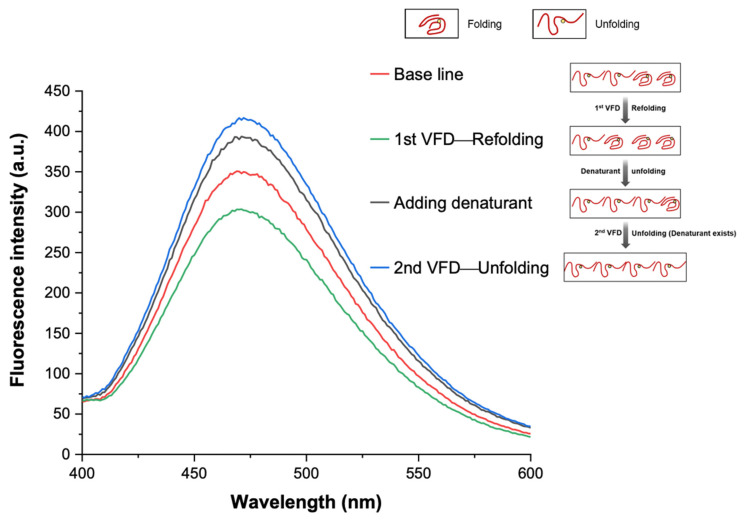
Refolding and unfolding under VFD processing, monitored by fluorescence. The fluorescence monitoring was carried out in PBS buffer with pH = 7.4. [TPE-MI] and [β-lactoglobulin] = 50 μM, [GuHCl] = 4 M. λ_ex_ = 350 nm.

We established that a combination of VFD and AIEgen technologies is effective for manipulating and monitoring protein refolding and unfolding, with the results shown in Figure 5. The fluorescence behaviour of the protein solution after 24 h denaturation and 3 h labelling by TPE-MI is set as the base line. At this condition, all the denaturant inside the solution has been consumed and most of the proteins are denatured. Thereafter VFD processing for 10 min resulted, as expected, in a decrease in fluorescence intensity at 470 nm dropped, with the VFD effectively refolding the unfolded proteins where less free-thiol in the proteins is available to bond with TPE-MI. Thus, the VFD can manipulate the protein refold and AIEgen can monitor this change, with an intensity change of ca. 14%. When a small amount of denaturant (~1 mL, 4 M) was added into the solution, the balance is broken, and more proteins become denatured with more free-thiol in the proteins available to bond with TPE-MI, thereby further increasing the fluorescence intensity. This result indicates that the β-sheet secondary structure of lactoglobulin is completely destabilized in the presence of the GuHCl ions. Finally, VFD processing was applied again for 10 min (at the same operating conditions—tilt angle 45°, ω 5000 rpm) which accelerated the denaturation, as determined by an increase in fluorescence intensity by ca. 6%.

## 4. Conclusions

We have developed a facile rapid and low costing vortex fluidic device (VFD) mediated method to control the refolded/unfolded protein state versus the renaturation/denaturation state for β-lactoglobulin. Importantly, the VFD mediated folding can be simultaneously monitored real time using a novel aggregation induced emission dye, TPE-MI. Moreover, the refolding and unfolding of the protein is performed simultaneously and the presence of denaturant and the ratio of folded/unfolded protein in different denaturation states determines which process is dominant. Overall, use of the VFD is effective in manipulating and accelerating the refolding/unfolding of a protein, which can be monitored by the AIEgen through fluorescence intensity changing. These findings demonstrate a promising strategy to control the dynamic process of protein folding/unfolding for practical applications.

## Data Availability

Not applicable.

## Sample Availability

Samples of the compounds are available from the authors.
